# Cultura de Seguranza do Paciente no Servido Médico de Urgencia: estudo transversal*

**DOI:** 10.15649/cuidarte.2531

**Published:** 2023-05-28

**Authors:** Virgilio Malundo-Joáo, Bruna Moreno-Dias, Marília Pilotto-de-Oliveira, Ana Maria Laus, Andrea Bernardes, Carmen Silvia-Gabriel

**Affiliations:** 1 . Universidade de Sáo Paulo, Ribeiráo Preto, SP, Brasil. Email: malundogido@yahoo.com.br Universidade de São Paulo Universidade de Sáo Paulo Ribeiráo Preto SP Brazil malundogido@yahoo.com.br; 2 . Universidade de Sáo Paulo, Ribeiráo Preto, SP, Brasil. Email: bruna.dias@usp.br Universidade de São Paulo Universidade de Sáo Paulo Ribeiráo Preto SP Brazil bruna.dias@usp.br; 3 . Prefeitura Municipal de Sertáozinho, Sertáozinho, SP, Brasil. Email: mariliapilotto@gmail.com Prefeitura Municipal de Sertãozinho Prefeitura Municipal de Sertáozinho Sertáozinho SP Brazil mariliapilotto@gmail.com; 4 . Universidade de Sáo Paulo, Ribeiráo Preto, SP, Brasil. Email: analaus@eerp.usp.br Universidade de São Paulo Universidade de Sáo Paulo Ribeiráo Preto SP Brazil analaus@eerp.usp.br; 5 . Universidade de Sáo Paulo, Ribeiráo Preto, SP, Brasil. Email: andreab@eerp.usp.br Universidade de São Paulo Universidade de Sáo Paulo Ribeiráo Preto SP Brazil andreab@eerp.usp.br; 6 . Universidade de Sáo Paulo, Ribeiráo Preto, SP, Brasil. Email: cgabriel@eerp.usp.br Universidade de São Paulo Universidade de Sáo Paulo Ribeiráo Preto SP Brazil cgabriel@eerp.usp.br

**Keywords:** Seguranza do Paciente, Equipe de Assistencia ao Paciente, Servidos Médicos de Emergencia, Assistencia Pré-Hospitalar, Estudos Transversais, Patient Safety, Patient Care Team, Emergency Medical Services, Prehospital Care, Cross Sectional Studies, Seguridad del Paciente, Grupo de Atención al Paciente, Servicios Médicos de Urgencia, Atención Prehospitalaria, Estudios Transversales

## Abstract

**Introdujo:**

Os problemas relacionados a seguranza do paciente no contexto pré-hospitalar sáo pouco explorados, porém essenciais, dada a vulnerabilidade para a ocorrencia de incidentes.

**Objetivo:**

Analisar o clima de seguranza do paciente na perspectiva da equipe multiprofissional que atua no Atendimento Pré-Hospitalar Móvel (APH).

**Materiais e Métodos:**

Estudo transversal, conduzido em um Atendimento Pré-Hospitalar Móvel. A coleta de dados foi realizada por meio do Safety Attitudes Questionnaire (SAQ), com amostragem por conveniencia e taxa de participado de 94,3% dos profissionais elegíveis. Empregou-se estatística descritiva e o teste Mann-Whitney para análise de dados.

**Resultados:**

Dentre os 151 profissionais participantes, predominaram aqueles do sexo masculino (54,6%), auxiliares e técnicos de enfermagem (42,0%), atuantes há 10 ou mais anos (61,0%), em atendimentos adultos e pediátricos (93,4%). O SAQ Total apresentou mediana de 70, indicando percepáo negativa dos participantes acerca da seguranza do paciente no APH. Os dominios com percepáo negativa foram: Clima de Seguranza, Reconhecimento de Estresse, Percepáo da Gestáo e Condióes de Trabalho; enquanto os dominios Clima de Trabalho em Equipe e Satisfago no Trabalho apresentaram percepáo positiva. Na análise comparativa entre os profissionais, foram observadas diferenas entre algumas categorias para os dominios Satisfago no Trabalho, Reconhecimento de Estresse e Condióes de Trabalho.

**Conclusoes:**

Este estudo apresenta as peculiaridades dos servidos de médicos de emergencia e a necessidade de sensibilizar profissionais e gestores acerca da temática seguranza do paciente, com vistas a melhor compreensáo do atual cenário e possibilidades de redudo de eventos adversos para a melhoria da assistencia ofertada.

## Abstract (en)

**Introduction:**

Problems related to patient safety in the pre-hospital context are little explored, but essential, given the vulnerability to incidents.

**Objective:**

To analyze the patient safety climate from the perspective of the multidisciplinary team that works in Mobile Pre-Hospital Care (PHC).

**Materials and Methods:**

Cross-sectional study, conducted in a Mobile Pre-Hospital Care. Data collection was performed through Safety attitudes Questionnaire (SAQ), with convenience sampling and participation rate of 94.3% of eligible professionals. Descriptive statistics and the Mann- Whitney test were used for data analysis.

**Results:**

Among the 151 participating professionals, males predominated (54.6%), nursing assistants and technicians (42.0%), working for 10 or more years (61.0%), in adult and pediatric care (93.4%). The SAQ Total had a median of 70, indicating a negative perception of the participants about patient safety in the APH. The domains with a negative perception were: Safety Climate, Stress Recognition, Management Perception and Working Conditions; while the Teamwork Climate and Job Satisfaction domains showed a positive perception. In the comparative analysis among professionals, differences were observed between some categories for the domains of Job Satisfaction, Stress Recognition and Working Conditions.

**Conclusions:**

This study presents the peculiarities of emergency medical services and the need to sensitize professionals and managers about patient safety, with a view to better understanding the current scenario and possibilities of reducing adverse events to improve the care offered.

## Resumen

**Introducción:**

Los problemas relacionados con la seguridad del paciente en el contexto prehospitalario son poco explorados, pero esenciales, dada la vulnerabilidad a incidentes.

**Objetivo:**

Analizar el clima de seguridad del paciente desde la perspectiva del equipo multidisciplinario que actúa en la Atención Prehospitalaria Móvil (APS).

**Materiales y Métodos:**

Estudio transversal, realizado en una Atención Prehospitalaria Móvil. La recolección de datos se realizó a través de Seguridad actitudes Cuestionario (SAQ), con muestreo por conveniencia y tasa de participación del 94,3% de los profesionales elegibles. Para el análisis de los datos se utilizó estadística descriptiva y la prueba de Mann-Whitney.

**Resultados:**

Entre los 151 profesionales participantes, predominaron los del sexo masculino (54,6%), auxiliares y técnicos de enfermería (42,0%), con 10 o más años de actuación (61,0%), en la atención de adultos y pediátrica (93,4%). El SAQ Total tuvo una mediana de 70, indicando una percepción negativa de los participantes sobre la seguridad del paciente en la APH. Los dominios con percepción negativa fueron: Clima de Seguridad, Reconocimiento del Estrés, Percepción de la Gestión y Condiciones de Trabajo; mientras que los dominios Clima de Trabajo en Equipo y Satisfacción Laboral mostraron una percepción positiva. En el análisis comparativo entre profesionales, se observaron diferencias entre algunas categorías para los dominios de Satisfacción en el Trabajo, Reconocimiento del Estrés y Condiciones de Trabajo.

**Conclusiones:**

Este estudio presenta las peculiaridades de los servicios médicos de emergencia y la necesidad de sensibilizar a los profesionales y gestores sobre la seguridad del paciente, con el fin de comprender mejor el escenario actual y las posibilidades de reducción de eventos adversos para mejorar la atención ofrecida.

## Introdujo

A seguranza do paciente tem sido objeto de interesse dos sistemas de saúde e dos pesquisadores, que, há cerca de 20 anos, tém se voltado para questóes da sua melhoria, no que tange a estrutura, processos, procedimentos, comportamentos e cultura, com vistas a reduzir riscos e danos evitáveis, bem como reduzir o impacto do erro, quando ele ocorre^1^.

O progresso na seguranza do paciente se deve aos relatórios e sistemas de aprendizagem, compromisso de lideranga, políticas e agóes práticas e, em especial, a proposito de uma cultura de seguranza positiva^1^.

A Cultura de Seguranga do Paciente (CSP), entendida como um conjunto de valores, atitudes, competéncias e comportamentos que determinam a seguranga do paciente^2^, é fundamental para conduzir melhorias e garantir avangos substanciais, buscando alinhamento entre conhecimento e implementagao de agóes^3^. Considerada como um importante componente estrutural dos servigos, a cultura CSP favorece a implantagao de práticas seguras e a diminuigao de incidentes e erros, que possam levar a qualquer tipo de dano prevenível ao paciente nos servigos de assisténcia a saúde, os eventos adversos^1^^,^^4^.

Dessa forma, investir em uma cultura organizacional, que habilite e priorize a seguranga, garantirá aos pacientes um cuidado seguro em todos os ambientes de assisténcia^3^.

Uma das formas de compreender a CSP se faz pela da mensuragao do clima de seguranga, que retrata a percepgao do estado de seguranga em determinado local e momento. Percepgóes mais positivas do clima de seguranga estao associadas a menor ocorréncia de incidentes e eventos adversos, além de resultados positivos em indicadores como tempo de permanéncia hospitalar, readmissao e mortalidade^5^.

Para mensuragao do clima de seguranga, o *Safety Attitudes Questionnaire* (SAQ) tem sido uma opgao de escolha, em fungao da facilidade de compreensao^6^. O SAQ foi desenvolvido por Sexton e colaborares, em 2006^7^, para aplicagao em diversas áreas assistenciais, como ambulatórios, setores de internagao, centro cirúrgico e unidades de terapia intensiva. O questionário permite a mensuragao das atitudes dos profissionais em seis domínios relacionados a seguranga do paciente, a saber: Clima de Seguranga, Clima de Trabalho em Equipe, Percepgóes da Gestao, Satisfagao no Trabalho, Condigóes de Trabalho e de Reconhecimento de Estresse^7^.

Ainda que a compreensao da CSP seja relevante em todos os servigos de saúde, observa-se que em ambientes extra-hospitalares este tema é pouco abordado. Especificamente nos servigos de Atendimento Pré-Hospitalar Móvel (APH), os problemas relacionados a seguranga do paciente sao pouco investigados e, consequentemente, subestimados^8^.

No ámbito da seguranga do paciente no APH, revisao integrativa da literatura aponta lacuna de estudos nesta área, ainda que este seja um ambiente que lida com pacientes críticos e tomada de decisao rápida, com impacto direto no desfecho do atendimento^9^. Complementarmente, a revisao destaca elementos como o conhecimento de protocolos, a comunicagao em equipe, a qualidade dos registros e as capacitagóes como possíveis temas para melhoria da qualidade nesses servigos^9^.

As atividades prestadas nos servidos de APH, destinadas as situagóes de urgencia e emergencia, sao entendidas como complexas, dinámicas, desafiadoras e estressantes, por este motivo, sao mais vulneráveis a ocorrencia de eventos adversos^10^. Tais servigos sao prestados por equipe multiprofissional e se caracterizam pela necessidade de atendimento ao paciente em um curto espago de tempo, em razao da condigao clínica do paciente, elevando os riscos a que estao expostos^11^.

A atengao as urgencias, no cenário brasileiro, tem se desenvolvido nas últimas duas décadas principalmente por meio da regulamentagao e implantagao da Política Nacional de Atengao as Urgencias e implantagao do Servigo de Atendimento Móvel de Urgencia (SAMU 192) e das Unidades de Pronto Atendimento (UPA); estratégias voltadas para organizagao de um sistema integrado de atengao as urgencias^12^.

Considerando que o SAMU é um importante componente da atengao as urgencias^12^ e que a seguranga do paciente depende de melhorias em todos os contextos e servigos de saúde, inclusive no atendimento pré-hospitalar^3^, especialmente porque as situagóes de urgencia e emergencia podem dificultar as agóes de seguranga, podendo resultar em comportamentos e em atitudes falhas no que tange as agóes de seguranga; esse estudo se volta para os profissionais do servigo de atendimento móvel de urgencia.

Compreender a cultura de seguranga deve ser o ponto de partida para iniciar o planejamento de agóes que garantam o cuidado de saúde seguro^13^, de modo que se constitua em uma ferramenta estratégica para instituigóes e gestores^14^. Adicionalmente, no ámbito da pesquisa, a elaboragao de estudos sobre a cultura, tal como enfatizado no Programa Nacional de Seguranga do Paciente, permitirá a produgao, sistematizagao e expansao do conhecimento da seguranga do paciente^15^.

A especificidade do atendimento prestado nos servigos médicos de urgencia e a imprescindibilidade da oferta de assistencia segura, apontam para a necessidade de compreender e conhecer a percepgao da equipe multiprofissional dos servigos de APH Móvel acerca da cultura de seguranga do paciente. Frente ao exposto, o objetivo deste estudo foi analisar o clima de seguranga do paciente na perspectiva da equipe multiprofissional que atua no atendimento pré-hospitalar móvel.

## Materiais e Método

Estudo transversal, de abordagem quantitativa, conduzido no servigo público de Atendimento Pré- Hospitalar Móvel do Servigo de Atendimento Móvel de Urgencia (SAMU-192) da cidade de Ribeirao Preto, Sao Paulo, Brasil.

O servigo opera sete dias por semana, em regime de 24 horas ininterruptas; tem em sua frota ativa um total de 17 ambuláncias, sendo duas de categoria D (unidade de suporte avangado), 12 de categoria B (unidade de suporte básico) e tres de categoria A (unidade de transporte simples).

A populagao do estudo foi constituída pelos profissionais que atuam nas unidades de suporte básico e avangado, composta por dez enfermeiros, cinco técnicos de enfermagem, 63 auxiliares de enfermagem, 25 médicos e 57 condutores de veículo de urgencia; totalizando 160 profissionais. Foram considerados elegíveis todos os profissionais atuantes no servigo, a excegao daqueles que estivessem de licenga saúde ou afastamento por tempo indeterminado, férias e aqueles que atuam em cargo de chefia. Amostra por conveniencia, com participagao 151 dos 160 profissionais elegíveis (94,3% da populagao); as nove perdas se deram em razao de recusa.

A coleta de dados foi realizada no período de julho a outubro de 2019. Os participantes foram abordados pessoalmente pelo pesquisador em seus locais de trabalho, nos turnos diurno e noturno, sendo considerada a disponibilidade de horário da instituido e dos participantes.

Para coleta de dados foi utilizada a versáo brasileira do Safety Attitudes Questionnaire (SAQ), traduzido e validado para o portugués. Trata-se de um instrumento autoaplicável, composto por duas partes, sendo que na primeira constam 41 itens distribuídos em 6 domínios, a saber: Clima de Trabalho em Equipe (CTE), Clima de Seguranza (CS), Satisfagáo no Trabalho (ST), Reconhecimento de Estresse (RE), Percepgáo da Gestáo (PG) e Condigóes de Trabalho (CT). Na segunda parte do instrumento, constam dados de caracterizado sociodemográfica, composta pelas variáveis: sexo, cargo, tempo na especialidade e atuagáo^16^.

O domínio Percepgáo da Gestáo, originalmente, é composto por duas partes, Administragáo da unidade e Administragáo do hospital^16^. Para efeito deste estudo, náo foram considerados os cinco itens relacionados a Administrado do hospital, uma vez que náo sáo aplicáveis ao cenário de estudo. Desta forma, foram considerados 36 itens e nos seis domínios do SAQ.

Para cada um dos itens, os participantes foram solicitados a responder baseados em uma escala de concordancia de 5 pontos, sendo que o escore varia de 0 a 100, em que zero é a pior percepgáo do clima de seguranga e 100 a melhor percepgáo. Para cada um dos domínios foram calculadas as médias, considerando válido para o cálculo, aqueles com preenchimento de, pelo menos, 50% dos itens do domínio. Para análise do valor final, SAQ total, os valores iguais ou superiores a 75 foram considerados como de forte concordancia em questóes de seguranga do paciente, classificados como atitude de seguranga positiva, enquanto valores inferiores a 75 foram classificados como atitude de seguranga negativa^16^.

Os dados coletados foram duplamente digitados em planilhas eletronicas no *software* Microsoft Excel 2020, com posterior verificagáo de consisténcia da digitagáo. Para composigáo do banco de dados e análise, foi utilizado o *software* IBM SPSS Statistics versáo 25.

Foi empregada estatística descritiva para caracterizagáo dos participantes e dos domínios do SAQ. Para análise da confiabilidade do questionário, foi aplicado o coeficiente alfa de Cronbach, que varia em uma escala de 0 a 1, sendo considerados ideais coeficientes superiores a 0,7^17^. A normalidade foi verificada por meio do teste de Shapiro-Wilk. O teste Kruskal-Wallis foi utilizado para comparagáo entre categorias profissionais; a escolha desse teste se deu em razáo do número de participantes em cada grupo de comparagáo e comportamento das variáveis, que náo apresentaram distribuigáo normal. Foi realizado o modelo de regressáo log-binomial para análise de fatores relacionados a atitude de seguranga. Foi considerando nível de significancia de 5% e intervalo de confianga de 95%.

Os preceitos éticos previstos na Resolugáo n° 466/12 do Conselho Nacional de Saúde foram assegurados. O estudo foi submetido a apreciagáo e aprovado pelo Comité de Ética em Pesquisa em 14 de maio de 2019, sob parecer número: 3.324.444.

## Resultados

Participaram deste estudo 151 dos 160 profissionais elegíveis; as nove perdas se deram em razáo de recusa e o estudo contou com taxa de resposta de 94,3%. Predominaram participantes do sexo masculino (54,6%) e o cargo de auxiliares e técnicos de enfermagem (42,0%). Os profissionais relataram atuagáo em APH móvel por período superior a 10 anos (61,0%) e atuagáo em atendimentos para adultos e pediatria (93,4%). Todos os participantes possuem vínculo empregatício efetivo, selecionados por meio de concurso público municipal (Tabela 1).

**Tabela 1:** Caracterizaáo dos participantes, segundo variáveis demográ cas e de trabalho (n=151). Ribeiráo Preto, Sáo Paulo, Brasil, 2021.

Variáveis	Frequencias	
	n	%
Genero		
Homem	87	57,6
Mulher	63	41,7
Nao informado	1	0,7
Cargo		
Técnico/Auxiliar de Enfermagem	64	42,0
Condutor de Veículo de Urgencia	53	35,1
Médico Assistencial	15	9,9
Enfermeiros	10	6,6
Médico Regulador	8	5,3
Médico Residente	1	0,7
Atuaao Principal		
Adulto	6	4,0
Adulto e Pediatria	141	93,4
Nao informado	4	2,6
Tempo na Especialidade		
Menos de 5 anos	20	13,2
5 a 10 anos	39	25,8
11 a 20 anos	62	41,1
21 anos ou mais	29	19,2
Nao informado	1	0,7

A confiabilidade do instrumento foi mensurada com a utilizado do alfa de Cronbach, que teve valor de 0,86 para o instrumento como um todo, SAQ Total; e demonstrou boa confiabilidade (valores superiores a 0,7) em quatro domínios (Tabela 2).

**Tabela 2:** Coeficiente alfa de Cronbach, segundo domínios do Questionário de Atitudes de Seguranza (n=151). Ribeiráo Preto, Sao Paulo, Brasil, 2021.

Domínio	Números de Itens	Números Válidos	Alfa de Cronbach
Clima de Trabalho em Equipe	6	151	0,486
Clima de Seguranza	7	151	0,582
Satisfaao no Trabalho	5	150	0,636
Reconhecimento de Estresse	4	151	0,770
Percepao da Gestao	6	148	0,758
Condioes de Trabalho	4	141	0,713
SAQ total	36	151	0,860

O SAQ Total apresentou mediana de 70 e intervalo interquartil (IIQ) de 16,3, indicando percepgáo geral negativa dos participantes acerca de questóes de seguranza do paciente. Ao observar os dominios destaca-se que dois apresentam percepgáo positiva, Clima de Trabalho em Equipe (75; 21) e Satisfago no Trabalho (95; 15). Os dominios com percepgáo negativa foram: Clima de Seguranza, Reconhecimento de Estresse, Percepgáo da Gestáo e Condigóes de Trabalho, sendo que o dominio percepgáo da Gestáo foi o que apresentou valores mais baixos (55,5; 32) (Tabela 3).

**Tabela 3:** Análise descritiva dos escores do questionário de atitudes de seguranza, segundo domínios (n=151). Ribeiráo Preto, Sáo Paulo, Brasil, 2021.

Domínio	Mediana	Intervalo interquartil	Mínimo - Máximo	p-valor*
Clima de Trabalho em Equipe	75	21	33,0 - 100	< ,001
Clima de Seguranza	68	22	29,0 - 100	0,005
Satisfago no Trabalho	95	15	45,0 - 100	< ,001
Reconhecimento de	63	46	0,0 - 100	< ,001
Estresse				
Percepao da Gestao	55,5	32	4,0 - 100	0,267
Condioes de Trabalho	67	42	0,0 - 100	< ,001
SAQ total	70	16,3	38,0 - 95,0	0,323

* Teste de Shapiro-Wilk.

Ao comparar as categorias profissionais (Tabela 4), o dominio Condigóes de Trabalho foi o único com diferenga significativa entre grupos (p = 0,010). De maneira geral, para todos os grupos observa-se maior pontuagáo no dominio Satisfagáo no Trabalho e menor pontuagáo para a Percepgáo da Gestáo. Na pontuagáo total, os enfermeiros apresentam menor pontuagáo, enquanto médicos reguladores apresentam maior pontuagáo.

**Tabela 4:** Comparado de escores obtidos por dominio entre as categorías pro ssionais (n = 151). Ribeirao Preto, Sao Paulo, Brasil, 202l.

Domínio	Enfermeiro	Médicos	Médico Regulador	Técnico e Auxiliar de Enfermagem	Condutor de Veículo de rgencia	p-valor*
Clima de Trabalho em Equipe	73 (23,5)	81 (14)	79 (16,5)	75 (20)	79 (18)	0,229
Clima de Seguranza	64,5 (34,3)	64 (23,5)	73 (42,8)	68 (22,8)	71 (29)	0,570
Satisfaao no Trabalho	92,5 (22,5)	77,5 (36,3)	95 (10)	95 (15)	95 (10)	0,172
Reconhecimento de Estresse	56,5 (28)	63 (25,3)	87,5 (16)	56 (39,5)	63 (50)	0,078
Percepao da Gestao	46 (23,5)	48 (30,8)	62,5 (32,5)	59 (28)	54 (36,3)	0,413
Condioes de Trabalho	46 (20,8)	54 (33,3)	71 (30,3)	75 (40)	67 (58)	0,010
SAQ total	63,8 (15,6)	67,5 (13,8)	71,3 (19,4)	67,5 (15)	70 (20)	0,422

*Teste Kruskal-Wallis.

Na Tabela 5 observam-se os fatores relacionados a atitude de seguranza, adotando-se a atitude de seguranza positiva a pontuagáo igual ou superior a 75 pontos. Neste modelo, observa-se que variáveis de género, tempo de atuagáo na especialidade e cargo náo tém associagáo com a atitude de seguranza.

**Tabela 5:** Modelo de regressao log-binomial de fatores relacionados a atitude de seguranza (n = 151). Ribeirao Preto, Sao Paulo, Brasil, 2021.

Variáveis	Positiva n (%)	Negativa n (%)	p-valor	Odds Ratio (IC 95%)
Genero				
Masculino	32 (36,78)	55 (63,22)		ref.
Feminino	18 (28,57)	45 (71,43)	0,928	10,45 (0,40 - 2,72)
Tempo na especialidade				
Menos de 5 anos	5 (25,00)	15 (75,00)		ref.
5 a 10 anos	15 (38,46)	24 (61,54)	0,341	18,37 (0,53 - 6,43)
11 a 20 anos	16 (25,81)	46 (74,19)	0,930	10,57 (0,31 - 3,61)
21 anos ou mais	13 (44,83)	16 (55,17)	0,224	22,44 (0,61 - 8,25)
Cargo				
Enfermeiros	1 (10,00)	9 (90,00)		ref.
Médicos	4 (25,00)	12 (75,00)	0,336	32,53 (0,29 - 36,05)
Médico regulador	3 (37,50)	5 (62,50)	0,255	43,80 (0,34 - 55,84)
Técnico/Auxiliar de Enfermagem	20 (31,25)	44 (68,75)	0,202	40,93 (0,47 - 35,72)
Condutor de Veículo de Urgencia	22 (41,51)	31 (58,49)	0,111	63,32 (0,65 - 61,37)

## Discussao

Os achados deste estudo apontam para uma atitude de seguranza negativa, uma vez que a mediana do SAQ foi inferior a 75 pontos.

Resultados semelhantes foram observados em outros estudos nacionais, em diferentes contextos, como em tres hospitais localizados no estado do Ceará^18^ e em tres Unidades de Terapia Intensiva (UTI) do Estado do Piauí^19^.

No contexto de situagóes de urgencia e emergencia, também foi observada a percepgáo negativa em uma unidade de emergencia de um hospital de grande porte no Estado de Sáo Paulo^10^ e em um hospital público de urgencia em Goiás^20^.

A despeito da percepgáo negativa no SAQ Total, foram observadas atitudes positivas nos domínios Clima de Trabalho em Equipe e Satisfagáo no Trabalho.

Em diversos estudos, a Satisfagáo no Trabalho tem sido apontada como domínio de atitude de seguranga positiva^10^^,^^18^^,^^21^. No estudo realizado nos hospitais do Ceará, este domínio foi o que obteve maior escore^18^, a maioria dos profissionais de unidade de emergencia demonstrou satisfagáo no trabalho^10^ e, em um hospital público de ensino do interior de Sáo Paulo, este foi o único domínio com percepgáo positiva sobre a atitude de seguranga^22^.

De forma similar, uma instituigáo de nivel terciário na Colombia, por meio da utilizagáo do Hospital Survey on Patient Safety Culture, identificou o trabalho em equipe e as dimensóes de aprendizagem organizacional e melhoria contínua como pontos fortes da cultura de seguranga dessa instituigáo^23^. Estudo realizado em dois hospitais da Arábia Saudita apontou uma correlagáo negativa significativa entre o número de EA e o clima de trabalho em equipe, satisfagáo no trabalho e condigóes de trabalho^24^.

Assim como o trabalho em equipe, a satisfagáo no trabalho é uma das características fundamentais para a consolidagáo de uma cultura positiva em qualquer ambiente de assistencia a Saúde^14^. Por outro lado, profissionais insatisfeitos apresentam elevadas taxas de rotatividade, com impacto negativo na seguranga do paciente^25^.

Entende-se que, o envolvimento dos trabalhadores de diversos setores e níveis nas discussóes acerca da seguranga do paciente promove a integragáo e a contribuigáo com a instituigáo, unindo esforgos e potencializando o surgimento de ideias e a realizagáo de atividades com um alto desempenho^26^, reforgando a compreensáo da responsabilidade coletiva e a consolidagáo da cultura de seguranga.

Tais achados, apontam para a compreensáo da ST e TE como pontos fortes do servigo de APH em estudo, indicando potencial positivo desses domínios para a melhoria de seguranga do paciente.

A percepgáo positiva para os domínios de Clima de Trabalho em Equipe e Satisfagáo no Trabalho; e a percepgáo negativa para os demais domínios (Clima de Seguranga, Reconhecimento de Estresse, Percepgáo da Gestáo e Condigóes de Trabalho) foram constatadas na percepgáo de profissionais de enfermagem de tres UTI do Estado do Piauí^19^.

No que concerne a percepgáo da gerencia, valores baixos também foram observados no contexto hospitalar^18^ e em uma unidade de emergencia^10^, indicando uma percepgáo da falta de foco da gerencia nas questóes atinentes a seguranza; as pontuagóes reduzidas neste dominio tem como possíveis fatores a possível instabilidade de vínculo empregatício ou o modelo de retaliagáo^10^^,^^18^. Em contrapartida, o apoio da gerencia e as condigóes de trabalho sáo fatores que ampliam o escopo de atividades e responsabilidades para a melhoria da seguranga do paciente^14^.

Os domínios Condigóes de Trabalho e Reconhecimento do Estresse, avaliados negativamente pelos participantes deste estudo, permeiam a percepgáo de atitudes de seguranga do paciente no contexto do APH. Sáo reconhecidos fatores que influenciam negativamente esses domínios: o desequilíbrio entre demanda de servigo e disponibilidade de recursos humanos ou materiais^9^, a exposigáo a violencias físicas, abusos verbais e assédios sexuais durante suas atividades laborais^27^, a ocorrencia de acidentes de trabalho e riscos ocupacionais^28^, além de questóes pertinentes a qualidade de vida, como dor, desconforto e fatores do ambiente físico, como condigáo climática e exposigáo a ruídos, poluigáo e transito^29^.

Adicionalmente, considera-se que os investimentos em infraestrutura, base para as condigóes de trabalho, sáo indispensáveis para se atingir e manter as condigóes mínimas de seguranga em instituigóes de alto risco^30^.

Os achados deste estudo para os domínios RE e CT refutam a percepgáo positiva de trabalhadores de saúde de um servigo médico de emergencia na Espanha. Ainda que no estudo espanhol tenham sido identificados fatores positivos relacionados ao número de profissionais, condigóes de trabalho, apoio do superior imediato e trabalho em equipe, os autores do estudo apontam para a necessidade de ampliar agóes educativas dos profissionais acerca da seguranga do paciente, além de sugerir a implantagáo de um sistema de notificagáo e registro de eventos adversos^31^.

Na análise comparativa entre os profissionais, foram observadas diferengas entre grupos apenas no domínio Condigóes de Trabalho. De maneira geral, os enfermeiros apresentam menor pontuagáo nos domínios e escore geral, enquanto médicos reguladores apresentam maior pontuagáo.

Em um hospital de ensino no interior de Sáo Paulo, ao comparar a percepgáo de seguranga de enfermeiros, técnicos e auxiliares de enfermagem, foram observadas diferengas para todos os domínios, exceto RE, com registro de pontuagóes superiores para os enfermeiros^22^.

Em trabalhos internacionais, em razáo das diferengas na composigáo da forga de trabalho em saúde, sáo mais recorrentes as comparagóes entre enfermeiros e médicos. Em hospitais albaneses foram observadas diferengas nas pontuagóes dos domínios trabalho em equipe, reconhecimento do estresse, satisfagáo no trabalho e condigóes de trabalho^6^; e, de fora similar, em hospitais da Arábia Saudita, os enfermeiros apresentaram percepgáo negativa no trabalho em equipe e os médicos nas condigóes de trabalho; o que sugere que médicos e enfermeiros parecem entender de forma diferente as atitudes de seguranga^24^.

Ainda que náo tenham sido identificados estudos no contexto do APH, voltados para a análise e a comparagáo da percepgáo de atitudes de seguranga na perspectiva da equipe multiprofissional, cabe enfatizar que os níveis mais elevados de seguranga e qualidade das instituigóes sáo alcangados por meio da criagáo de um ambiente de apoio para toda a equipe envolvida na assistencia a saúde^5^.

Pesquisas sobre a temática de Cultura de Seguranza do Paciente tém sido desenvolvidas, majoritariamente, em ambientes hospitalares, sendo necessário avanzar as análises para o contexto extra-hospitalar. O instrumento utilizado neste estudo tem sido aplicado em diferentes países e contextos, apesar de possuir itens direcionados ao contexto hospitalar, o que pode ser considerado como uma limitagáo do estudo. Adicionalmente, entende-se como limitagáo os valores moderados do Alfa de Cronbach nos dominios "Clima de Trabalho em Equipe" e "Clima de Seguranza", no entanto, tendo em vista o valor obtido no escore total, entende-se que a consisténcia interna do instrumento seja válida para esta amostra. Apesar das limitagóes apresentadas, entende-se que os achados apresentados fornecem subsidios para identificagáo de fatores positivos e pontos de melhoria no atendimento pré-hospitalar móvel.

Este estudo contribui com pesquisadores, profissionais e gestores, na medida em que considera cenário extra-hospitalar e todas as categorias profissionais envolvidas no atendimento Pré-Hospitalar Móvel; além de identificar os dominios e as categorias profissionais em que sáo possiveis melhorias relacionadas a seguranza do paciente.

## Conclusao

Este estudo se configura como inédito no contexto dos servigos médicos de emergéncia, cenário em que se observou a percepgáo negativa em relagáo ao clima de seguranga do paciente, na perspectiva da equipe multiprofissional.

Ainda que a avaliagáo dos dominios de Clima de Trabalho em Equipe e Satisfagáo no Trabalho tenha se mostrado positiva, há pontos de melhoria nos demais dominios, a saber: Clima de Seguranga, Reconhecimento de Estresse, Percepgáo da Gestáo e Condigóes de Trabalho.

Na análise comparativa entre os profissionais, foram observadas diferengas entre algumas categorias para os dominios Satisfagáo no Trabalho, Reconhecimento de Estresse e Condigóes de Trabalho. Tais achados apontam para necessidade maior envolvimento de todas as categorias profissionais em temáticas pertinentes a seguranga do paciente.

O estudo apresenta as peculiaridades deste tipo de servigo de saúde e a necessidade de sensibilizar os profissionais e os gestores acerca da temática seguranga do paciente, com vistas a melhor compreensáo do atual cenário e possibilidades de redugáo de eventos adversos e melhoria da assisténcia a saúde.
